# Supplementary material for: Genome-wide CRISPR Screens in T Helper Cells Reveal Pervasive Crosstalk between Activation and Differentiation
Source: Cell. 2019 Feb 7;176(4):882–896.e18. doi: 10.1016/j.cell.2018.11.044 (PMC6370901; doi:10.1016/j.cell.2018.11.044)
Supplement: Data S2. Processed Data from All the Steps of the Analysis, Related to Figure 1 [file mmc2.zip › supplemental data/motif analysis/Stat6_SRX021632_homer/homerResults/motif7.similar.html]

motif7

## Information for motif7

C
T
G
A
C
T
A
G
C
A
T
G
C
T
A
G
A
C
T
G
A
C
T
G
G
A
T
C
C
T
A
G
A
C
T
G
C
T
A
G
T
C
A
G
G
T
A
C
  
Reverse Opposite:  

C
A
T
G
A
G
T
C
A
G
T
C
G
T
A
C
A
G
T
C
C
T
A
G
A
G
T
C
A
G
T
C
A
G
T
C
G
T
A
C
G
A
T
C
G
A
C
T
  

|  |  |
| --- | --- |
| p-value: | 1e-458 |
| log p-value: | -1.056e+03 |
| Information Content per bp: | 1.746 |
| Number of Target Sequences with motif | 4322.0 |
| Percentage of Target Sequences with motif | 17.10% |
| Number of Background Sequences with motif | 1970.2 |
| Percentage of Background Sequences with motif | 8.16% |
| Average Position of motif in Targets | 102.0 +/- 54.0bp |
| Average Position of motif in Background | 89.6 +/- 58.8bp |
| Strand Bias (log2 ratio + to - strand density) | -0.0 |
| Multiplicity (# of sites on avg that occur together) | 1.38 |
| Motif File: | file (matrix) reverse opposite |

### Similar de novo motifs found

|  |  |  |  |  |  |  |  |
| --- | --- | --- | --- | --- | --- | --- | --- |
| Rank | Match Score | Redundant Motif | P-value | log P-value | % of Targets | % of Background | Motif file |
| 1 | 0.932 | T C A G C T A G T A G C A G T C G T A C A G T C C T A G A G T C A G T C A G T C | 1e-431 | -993.143496 | 17.34% | 8.55% | motif file (matrix) |
| 2 | 0.771 | T C A G C A G T C T A G A T C G A C T G G T A C C T A G A C T G C T G A T C G A | 1e-349 | -805.233737 | 17.80% | 9.61% | motif file (matrix) |
| 3 | 0.911 | C T A G A G T C A G T C G T A C A G T C C T A G A G T C A G T C | 1e-254 | -585.326420 | 16.76% | 9.82% | motif file (matrix) |
| 4 | 0.715 | C G T A A C T G A C G T A C T G A C T G A C T G G T C A C T A G A C T G C T G A C T A G C T A G | 1e-156 | -361.429567 | 4.87% | 2.07% | motif file (matrix) |
